# A Pollution Model Upgrade? Incorporating the Local Scale in National Models

**DOI:** 10.1289/ehp.119-a355a

**Published:** 2011-08-01

**Authors:** Bob Weinhold

**Affiliations:** Bob Weinhold, MA, has covered environmental health issues for numerous outlets since 1996. He is a member of the Society of Environmental Journalists.

Networks of fixed-site air monitors form the backbone of current efforts to predict health effects of air pollution. But there aren’t enough monitors on the ground to accurately account for local variations in ambient concentrations of air pollutants, even for those pollutants considered of most concern in many countries, such as fine particulate matter (PM_2.5_). Experts have long struggled to develop better predictive models so epidemiologic studies can be made more accurate and affordable, and so health surveillance, policies, and regulations can be made more effective. A team of Canadian and U.S. researchers says it has made some incremental advances in this predictive science [***EHP* 119(8):1123–1129; Hystad et al.**]. The researchers are using the results of their new study as part of the larger Carex Canada project to estimate cancer risks associated with known and suspected environmental and occupational carcinogens.

The researchers assembled readily available data from the National Air Pollution Surveillance monitoring network in Canada and devised models to predict ambient concentrations of five pollutants at a national scale while capturing within-city pollution variability. The five models generated results similar to those from models typically used for regional or city areas. They were able to predict 73% of the variability in readings taken at nitrogen dioxide (NO_2_) monitors, 68% of the variability for 1,3-butadiene, 67% of that for ethylbenzene, 62% of that for benzene, and 46% of that for PM_2.5_.

The NO_2_ and benzene model predictions were compared against predictions generated independently for seven cities using land-use regression (LUR) models keyed to numerous monitors in each city. The NO_2_ model predicted 43% of the variability in readings predicted by LUR models, compared with 18% using the common technique of simply interpolating between monitors using factors such as distance from the monitor, weather patterns, and known pollution sources. The within-city benzene model captured only 16% of variability predicted by LUR models, compared with 11% using interpolation.

Predictions from the five models were influenced most strongly by satellite data for PM_2.5_ and NO_2_, vehicle emissions (represented by road type and location), industry emissions (from large point sources), and population density. Other factors in the current models included land use type, small industry point sources, railroad length, elevation, temperature, precipitation, and generalized predictive gradients representing dispersion from specific sources.

Other input might substantially improve the models if it were readily available and covered appropriate time intervals and geographic areas. Such input could include more monitoring data for these and other toxics, more detailed dispersion predictions, and emissions data for area sources such as wildfires and wood burning.

